# Parametric Reconstruction of Glass Fiber-reinforced Polymer Composites from X-ray Projection Data—A Simulation Study

**DOI:** 10.1007/s10921-018-0514-0

**Published:** 2018-07-30

**Authors:** Tim Elberfeld, Jan De Beenhouwer, Arnold J. den Dekker, Christoph Heinzl, Jan Sijbers

**Affiliations:** 1 imec-VisionLab, Department of Physics, University of Antwerp, Universiteitsplein 1, 2610 Antwerpen, Belgium; 2 Delft Center for Systems and Control, Delft University of Technology, Mekelweg 2, 2628 CD Delft, The Netherlands; 3 Research Group X-Ray Computed Tomography, University of Applied Sciences Upper Austria, Stelzhamerstrasse 23, 4600 Wels, Austria

**Keywords:** $\upmu$CT, Materials science, Glass fiber reinforced polymer, GFRP, Parametric model, Tomography, Modeling of micro-structures

## Abstract

We present a new approach to estimate geometry parameters of glass fibers in glass fiber-reinforced polymers from simulated X-ray micro-computed tomography scans. Traditionally, these parameters are estimated using a multi-step procedure including image reconstruction, pre-processing, segmentation and analysis of features of interest. Each step in this chain introduces errors that propagate through the pipeline and impair the accuracy of the estimated parameters. In the approach presented in this paper, we reconstruct volumes from a low number of projection angles using an iterative reconstruction technique and then estimate position, direction and length of the contained fibers incorporating *a priori* knowledge about their shape, modeled as a geometric representation, which is then optimized. Using simulation experiments, we show that our method can estimate those representations even in presence of noisy data and only very few projection angles available.

## Introduction

Advanced composites such as glass fiber-reinforced polymers (GFRP) integrate essential features for future materials such as formability, low weight, high tensile and compressive strength and cost-effectiveness [1], thus allowing for tailored components in many different industries. Composites typically consist of a matrix component (e.g., resin matrix) and a reinforcement component (e.g., glass fibers) to achieve specific mechanical properties. X-ray micro-computed tomography ($\upmu$CT) is an imaging method to study the internal structure of those composites in a nondestructive way and with high spatial resolution in the micro-scale. The resulting volumetric image is then further processed to characterize features, such as the fiber direction or spatial distribution of the fibers, which have an influence on the mechanical properties of the materials. Current methods to characterize the structural properties of GFRP from high resolution $\upmu$CT images rely on a sequential work flow comprised of volumetric reconstruction from a large number of projections (typically > 1000) and subsequent fiber segmentation and image analysis [2–6].

The reconstructed image quality depends on several parameters, such as the number of projections and the detector resolution, as well as the acquisition geometry. Since the accuracy of the identification of fibers in the volume heavily depends on the quality of the reconstruction, a long acquisition time is typically required. Additionally, parameters within the work flow from reconstruction to individual object characterization are typically determined in an empirical way, relying mainly on the experience of researchers. That is, many parameters have to be set manually or semiautomatically, in several steps of the work flow, which may introduce additional errors. Finally, because the conventional work flow is unidirectional, any error that occurs in one of the steps will propagate through the whole pipeline.

One big source of errors is that many methods require human intervention, making them non-deterministic and very reliant on researcher experience. To provide more automated solutions for single fiber extraction and measurement of fiber quantities, many approaches have been introduced lately. Many of them involve extracting the center lines of the fibers. The individual method of extracting the center lines and the use of the data differs in the approaches, though. Emerson et al. [7] use a dictionary learning approach to extract the centers of very low resolution fibers slice by slice, relying on the unidirectional fiber direction distribution of their datasets. Pinter et al. [8] use the local Eigenvalues and a circular voting approach. Huang et al. [9] use skeletonization to extract the center lines.

In our approach, we exploit our prior knowledge that the volume to be reconstructed contains fibers of a known shape. To that end, the fibers are modeled as cylinders whose parameters are estimated by fitting a model to the measured projection data, minimizing the projection distance. Initial values of the parameters are obtained from a first reconstruction of the volume using a conventional Algebraic Reconstruction Technique (ART), followed by a template matching approach similar to the one presented in [10]. A similar model-based approach was already implemented to reconstruct the crystal lattice of a gold nanoparticle at atomic resolution from electron tomography data [11].

The paper is structured in the following way: In Sect. 2 the methods and idea behind the algorithm are described. Section 3 deals with how the synthetic test data was generated and which experiments were performed. Subsequently, the results are summarized in Sect. 4 and a short summary of what we intend to add to our method in the future is given. We then conclude our findings in Sect. 6.

## Methods

The presented algorithm makes use of X-ray projection data simulated using the ASTRA toolbox framework [12]. Images in CT are reconstructed from projection data, which is acquired by measuring the intensities of the X-rays after they have passed through the sample, which attenuates the radiation. The measured intensity is related to the attenuation coefficients of the different materials in the sample by the equation

1 $$\begin{aligned} I(s) = \int \limits _{0}^{E_\mathrm{max}} I_{0}(E) \exp \left( -\int \limits ^{L}_{0}\mu (E, \eta ) d\eta \right) dE, \end{aligned}$$

where $I_{0}(E)$ is the incident beam intensity for a given energy, $\mu$ the energy dependent attenuation coefficient of the material in function of the distance *s* the X-ray travels inside a material and *I*(*s*) is the measured intensity on the detector depending on the running length through the object that is being imaged [13, 14]. In what follows, we assume monochromatic X-rays, simplifying the equation to the Lambert-Beer law. From a number of X-ray projections described by Eq. (1) acquired from several angles by either rotating the X-ray source and detector or the sample, a volumetric image can be reconstructed using different methods. In this paper we make use of ARTs, more specifically the well-known Simultaneous Iterative Reconstruction Technique (SIRT) algorithm [15].

### Parametric Reconstruction Algorithm

Using the projection images our algorithm, which in the following will be called parametric reconstruction (PARE), starts from an initial estimate of the reconstructed volume, obtained by performing a chosen number of iterations SIRT. Afterwards, we estimate the center position, direction and length of the fibers. This information is used to build a list of rigid cylinders, representing each fiber in the volume, that was detected by extracting the center line using a template matching approach. The template matching approach with a different template was previously used by Zauner et al. [10].

The cylindrical fiber model has seven parameters. The first three are *x*, *y* and *z* components of the centroid position. The subsequent two are the spherical coordinates $(\theta , \phi )$ of the direction unit vector of the fiber’s axis. The last two parameter are the fiber’s length *l* and radius *r*. Because the manufacturing process for glass fibers allows for very precisely adjustable radii [1], the fiber radius is assumed to be constant.

**Fig. 1:**
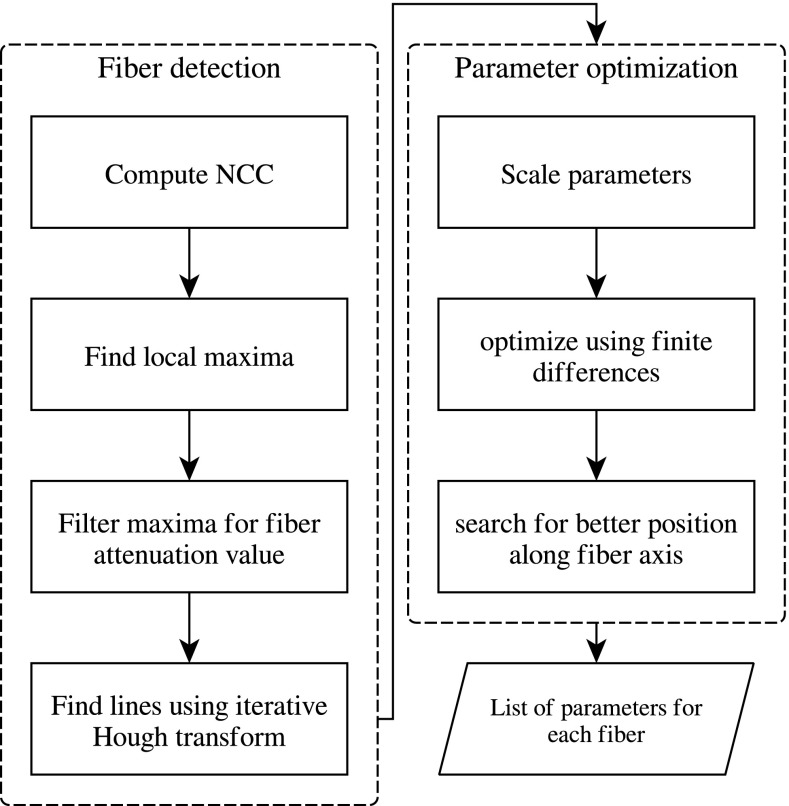
Flow chart of the fiber detection and parameter optimization steps

The estimation of the cylinder parameters consists of three main steps, which will be described in further detail in the following subsections. The first step is the overall detection of fibers in the current state of the reconstruction (Sect. 2.2). After detection, we obtain a first estimate of the parametric representations and then refine them by using a projection matching approach similar to the one presented in [16] (Sects. 2.3 and 2.4). A flowchart of the procedure is shown in Fig. 1.

### Segmentation of the Fiber System

To find voxels that are part of a fiber, the fibers need to be segmented from the background. As a first step of the segmentation, we compute the template matching, or more specifically the *normalized cross correlation* $C_{\text {n}}$ (NCC) of the reconstructed volume *I* with an isotropic 3D Gaussian template *G*

2 $$\begin{aligned} C_\mathrm{n}(\mathbf {u}) = \frac{\sum \limits _{\mathbf {x}} \left[ I(\mathbf {x})- \overline{I}_{\mathbf {u}} \right] \left[ G(\mathbf {x} - \mathbf {u}) - \overline{G}\right] }{\sqrt{\sum \limits _{\mathbf {x}} \left[ I(\mathbf {x})-\overline{I}_{\mathbf {u}}\right] ^2\sum \limits _{\mathbf {x}}\left[ G(\mathbf {x}- \mathbf {u})-\overline{G}\right] ^2}}, \end{aligned}$$

where $\mathbf {x}$ and $\mathbf {u}$ are the image and template coordinates respectively. The quantity $\overline{I}_{\mathbf {u}}$ is the local mean within the region of the image covered by the template and $\overline{G}$ is the mean intensity of G.

The algorithm used to compute Eq. (2) is an efficient implementation of the NCC introduced in [17]. The Gaussian template has a standard deviation dependent on the radius of the fibers using the definition of full-width at half-maximum

3 $$\begin{aligned} \sigma = \frac{r_{\text {fiber}}}{2 \sqrt{2 \ln 2}}, \end{aligned}$$

which then gives the following formula for the Gaussian template

4 $$\begin{aligned} G(\mathbf {x}) = \exp \left( -\frac{4\ln 2\left||\mathbf {x} \right||^2}{r_\mathrm{fiber}^2}\right) . \end{aligned}$$

We exploit the fact that the NCC has its highest values in the location of the center line of the fiber, which will be used to detect the direction and centroid in the following section. A template matching using a solid sphere has been shown to work as well [10].

**Fig. 2:**
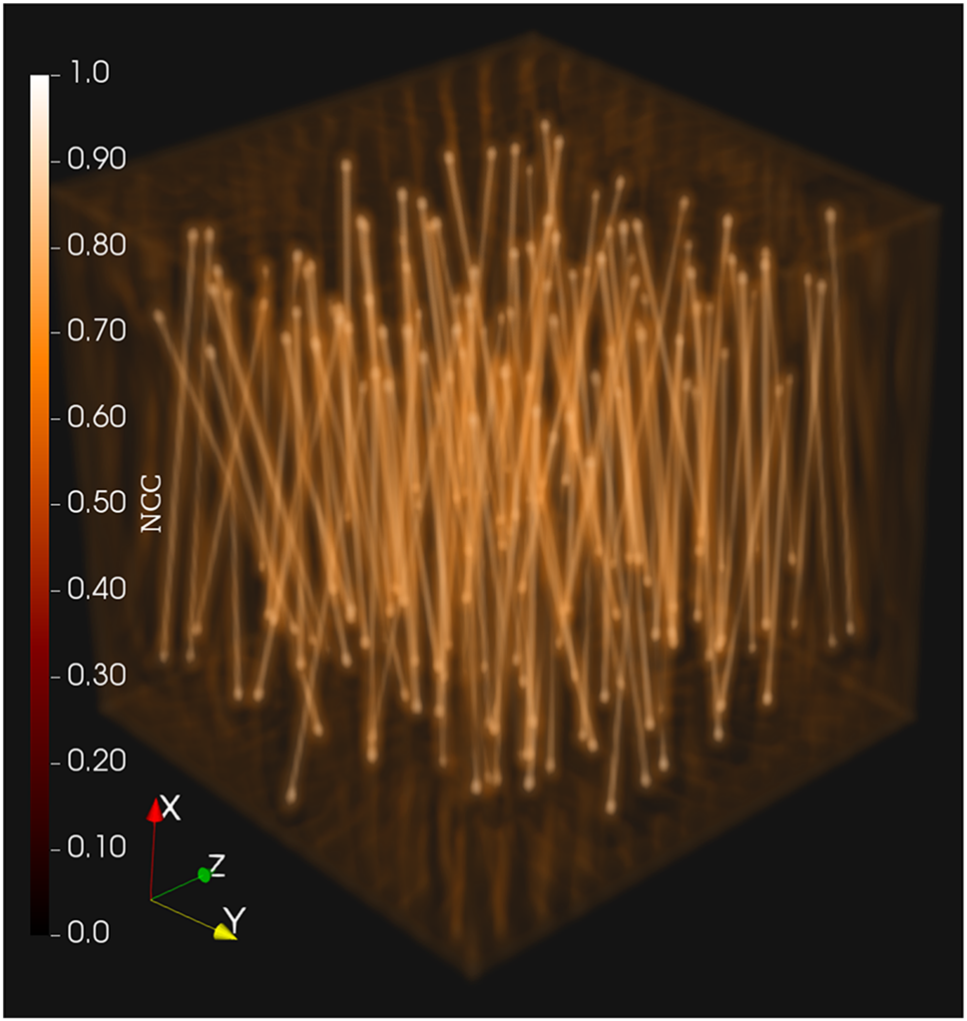
Result of applying the NCC to a reconstructed volume containing fibers (phantom A). It can clearly be seen that the fibers are enhanced in the resulting volume. The color bar serves as orientation, but values below 0.55 are not rendered and values between 0.55 and 0.79 are given a transparency value for better visibility (Color figure online)

With the NCC volume, we then proceed to detecting the fiber center line. For that, we first threshold $C_\mathrm{n}$

5 $$\begin{aligned} C_{\text {n,t}}({\mathbf {x}}) = \left\{ \begin{array}{lr} C_{\text {n}}({\mathbf {x}}), &{} \text {if } C_{\text {n}}({\mathbf {x}}) \ge t \max \limits _{{\mathbf {x}}} C_{\text {n}} ({\mathbf {x}})\\ 0, &{} \text {otherwise} \end{array} \right. , \end{aligned}$$

where all voxels lower than a threshold depending on the highest peak are set to zero, but the values above or equal to the threshold are kept. The value $t \in [0, 1]$ has to be adjusted to the particular image, as the range of the correlation values in $C_n$ will vary depending on the noise level in the image. The value of *t* gives a percentage of the maximum intensity in the NCC. For the simulations in the experiments section, a value of $t = 0.85$ was used. In Fig. 2 the result of the NCC on a sample phantom with a Gaussian according to Eq. (4) is visualized. It is clear that the fibers give the highest intensity in the resulting volume and that the center line is clearly visible.

We then obtain the local maxima in $C_\mathrm{n, t}$ in the region of the 26-neighborhood around a given voxel. The voxel values of the resulting maxima are then checked in the reconstruction, to make sure that all maxima are close to the attenuation value of the fibers. If the attenuation value is close to the expected fiber attenuation $\mu _{\text {fiber}}$ within a relative tolerance of $\pm \,25\%$, the point is marked as a fiber point and refined further by computing the center of mass using the 26-neighborhood of the voxel.

**Fig. 3:**
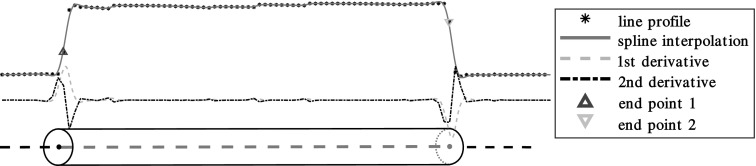
Line profile when sampling along a fiber in a noiseless volume. Shown is a measured line profile (marked with $*$), its spline interpolation (solid, red line) with 1st and 2nd derivative (orange dashed and purple, dash-dot lines, respectively) as well as the inflection points (brown, up-facing and yellow, down-facing triangles) (Color figure online)

### Fiber Estimation Initialization

From the filtered maxima of the NCC, an initial estimate of each fiber’s direction and centroid is computed using the iterative Hough Transform algorithm for 3D line segments proposed by Dalitz et al. [18].

This algorithm differs slightly from the traditional Hough transform. The voting accumulator uses a four parameter representation of the line [19]. The algorithm first performs the standard Hough transform algorithm on the point cloud and determines the parameters of one line using the highest peak in the accumulator. Next it finds all points in the point cloud that lie close to the detected line and computes a least square fit of those points to get a better estimate of the line. The points close to the fitted line are removed. After this procedure, the standard Hough transform is computed on the remaining points and the above process is repeated until there are either not enough points to compute the Hough transform anymore, or the specified number of lines were detected. If this limit is not specified, the algorithm runs until the first condition is met. In this paper we use the Hough transform with a minimum of three points, but we do not specify a limit on the number of lines to be detected.

The angular accuracy of the iterative Hough Transform algorithm is limited by the sampling of directions and relies on the iterative subdivision of an icosahedron’s faces into triangles. As a trade off between accuracy and speed, we chose 5 subdivisions steps, which yields 5121 samples with an average spacing of $2^{\circ }$ [20]. As a first estimate of the direction, we consider this sufficiently accurate. The lines are parametrized in the form

6 $$\begin{aligned} \mathbf {x} = \mathbf {x}_0 + s \mathbf {d}, \end{aligned}$$

with $\mathbf {x}_0$, the position vector and $\mathbf {d}$, the direction unit vector. With this initial estimate we only obtain five of the six parameters we want to optimize, namely centroid position and direction unit vector.

To also obtain an initial estimate of the length, we use an edge detection on a line profile through the newly estimated fiber axis. This is done using a 3-dimensional version of Bresenham’s line algorithm for line voxelization on a grid [21]. To make the detection more robust, we first apply a median filter to remove high frequency noise while preserving the edge in the signal and then smooth the line profile by a Gaussian filter with a standard deviation of $r_{\text {fiber}}$ voxels. Then the inflection points on either side of the line profile are computed by interpolating with B-splines and then finding the roots of the 2nd derivative with the highest slope in the 1st derivative [22]. Candidate inflection points are only considered if the slope has a higher value than a certain threshold, which should be chosen in relation to the value range between $\mu _{\text {fiber}}$ and $\mu _{\text {polymer}}$, which are the attenuation values for the fiber and the polymer matrix respectively. We chose $\mu _\mathrm{polymer} + 0.3 (\mu _\mathrm{fiber} - \mu _\mathrm{polymer})$. An illustration of this process with the line profile through a fiber and the interpolation with its derivatives is shown in Fig. 3.

To obtain the end point positions, the two nearest whole voxel points to the detected points on the Bresenham line are linearly interpolated depending on the fraction of the inflection point’s coordinate in the line profile. The initial estimate of the length is then the Euclidean distance of the two resulting end points.

### Optimization of the Fiber Parameters

With the parameter estimates from the iterative Hough transform and our length estimation as an initial starting point we now formulate the problem as an optimization of the fiber parameters. We optimize for the components for centroid position coordinates

$$\begin{aligned} {\mathbf {p}}_\mathbf{x}&= (p_{x,1}, \ldots , p_{x,N}),\\ {\mathbf {p}}_\mathbf{y}&= (p_{y,1}, \ldots , p_{y,N}),\\ {\mathbf {p}}_\mathbf{z}&= (p_{z,1}, \ldots , p_{z,N}), \end{aligned}$$

spherical coordinates of the direction unit vector

$$\begin{aligned} \mathbf {a}_{\varvec{\theta }}&= (a_{\theta ,1}, \ldots , a_{\theta ,N}),\\ \mathbf {a}_{\varvec{\phi }}&= (a_{\phi ,1}, \ldots , a_{\phi ,N}), \end{aligned}$$

and the length

$$\begin{aligned} \mathbf {l}&= (l_{1}, \ldots , l_{N}), \end{aligned}$$

for all *N* detected and estimated fibers. We then combine those into a fiber parameter vector ${\varvec{\xi }} = (\mathbf {p_x},\mathbf {p_y},\mathbf {p_z}, \mathbf {a}_{\varvec{\theta }}, \mathbf {a}_{\varvec{\phi }}, \mathbf {l})$ and set up a system of linear equations similar to the notation in [16]

7 $$\begin{aligned} {\mathbf {W}}{\mathbf {x}}({\varvec{\xi }}) = {\mathbf {p}}, \end{aligned}$$

where ${\mathbf {W}}$ is the projection matrix describing the forward projection with a fixed geometry and ${\mathbf {p}}$ is the measured projection data. The volume ${\mathbf {x}}$, that we want to reconstruct, is defined in function of the fiber parameters to be estimated. With that we can pose the optimization of the fibers as the minimization problem

8 $$\begin{aligned} \arg \min \limits _{{\varvec{\xi }}}\vert \vert {\mathbf {W}}{\mathbf {x}}({\varvec{\xi }}) - {\mathbf {p}}\vert \vert ^2. \end{aligned}$$

which minimizes the projection distance of the forward projection of the estimated volume to the measured projection data [16].

To initialize each fiber parameter vector estimate, we use the data retrieved from the Hough transform in the previous step and the length computed from the detected end points. With this initial estimate, we first scale the parameter ranges to the interval [0, 1] in each of the coordinates in our parameter space to make the method numerically more stable. The center of the interval, 0.5, is the initial estimate of each parameter $\xi _{i}$ and the outer boundaries are given by $\xi _{i}\pm \triangle _{i}$.

The values for $\triangle _{i}$ were chosen empirically to be 5 voxels (vx) for the position parameters $p_{x,i}, p_{y,i}, p_{z,i}$, for $a_{\theta ,i}$ and $a_{\phi ,i}$ $\triangle _{i}$ is three times the spacing between two samples in the Hough accumulator and for $l_{i}$ the value is 10vx. We also pre-initialize the volume ${\mathbf {x}} ({\varvec{\xi }})$ that we want to estimate. Given a fiber we want to optimize, we fix the $N-1$ remaining estimates of the fibers and voxelize them on a regular lattice grid that matches the assumed resolution of the fibers. We then systematically vary the parameters of the fiber to optimize and voxelize the resulting fiber into the same volume. This volume is then forward projected using the ASTRA toolbox [12] and the resulting projections are compared to the measured data.

The parameters for any given new estimate in an iteration *j* are computed with an estimation of the gradient $\widetilde{\nabla }_{j}$ using finite differences [23]. To that end, $\widetilde{\nabla } _{j}$ is first initialized to zero and then each parameter varied $\pm \delta$, where the initial $\delta =0.2$. If the projection error or projection distance $p_j$ of either one of the new values for the current parameter is lower or equal to the projection error of the previous best estimate, the difference between the two values is set as the value of $\widetilde{\nabla }_{j}$ for that parameter. If the projection error is *not* lower or equal, the gradient vector is assumed to be 0 for that particular parameter. After repeating this step for each parameter, $\widetilde{\nabla }_{j}$ is normalized to unit length and then used to compute a new estimate for the current fiber $\xi _{i} = (\widetilde{p}_{x,i}, \widetilde{p}_{y,i}, \widetilde{p}_{z,i}, \widetilde{a}_{\theta ,i}, \widetilde{a}_{\phi ,i}, \widetilde{l}_{i})$ as follows:

9 $$\begin{aligned} \xi _{i, \mathrm{new}} = \xi _{i} + \widetilde{\nabla }_{j} \delta . \end{aligned}$$

**Fig. 4:**
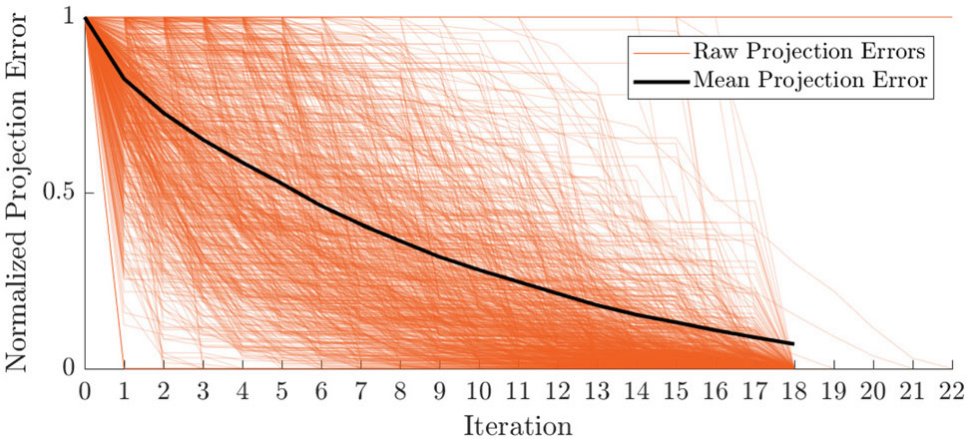
Normalized projection error as a function of the number of optimization iterations. Each thin, orange line corresponds to a single fiber. As the projection error varies drastically, the errors were normalized, so that the convergence can be compared (Color figure online)

The projection error for the new estimate $\xi _{i, \mathrm{new}}$ is then computed. If the error is lower or equal to the error of the previous estimate, $\xi _{i, \mathrm{new}}$ is taken as the new estimate of the fiber. If the error is not lower, the delta value is decreased to $75\%$ of its current value. This process is repeated for a minimum of $n_\mathrm{min}$ times and a maximum of $n_\mathrm{max}$ times. In this paper we use $n_\mathrm{min} = 18$ and $n_\mathrm{max} = 35$, both of which were chosen empirically. The iteration stops either at the upper limit of repetitions or if the rate of change of the error, defined as

10 $$\begin{aligned} \rho _j = 1 - \frac{p_{j}}{p_{j + 1}}. \end{aligned}$$

is lower than a threshold of 0.001. In Fig. 4, the normalized projection errors of 907 fibers is shown over the number of iterations. The maximum number of iterations is never reached and the error either stagnates or goes down, which shows that the fiber estimates either converge to a better solution or do not improve, if the initial estimate happens to already be good. Iteration 0 is the error for the initial estimate.

### Fiber Voxelization on a Cubic Lattice Grid

To estimate the volume ${\mathbf {x}}({\varvec{\xi }})$, we voxelize the current fiber estimates into a volume of the same size as the reconstruction. To that end, we sample the equation of a cylinder aligned with the *x*-axis with radius $r_{\text {fiber}}$ and the estimated length $l_{\text {fiber}}$

11 $$\begin{aligned} c(x,y,z) = \left\{ \begin{array}{lr} \mu _{\text {fiber}}, &{} \text {if } y^{2} + z^{2} \le r_{\text {fiber}}^2 \text { and } \vert x \vert < \frac{l_{\text {fiber}}}{2}\\ \mu _{\text {polymer}}, &{} \text {otherwise} \end{array} \right. \end{aligned}$$

To place the fiber in the estimated position along the estimated direction, we voxelize *c*(*x*, *y*, *z*) on a regular grid which we then transform such that the x-axis is directed along the fiber direction vector and the fiber centroid is located in the origin. We local-adaptively subsample voxels that would be set to $\mu _\mathrm{fiber}$, such that the voxel is subdivided *n* times in each coordinate direction, resulting in $n^3$ sample points per voxel. The values of each of those sub-voxels are then computed using Eq. (11). The values obtained that way are averaged and assigned to the corresponding voxel, thus aliasing the fiber borders.

The voxelization is also designed in a way that it will only replace the values that are in close proximity to the fiber, so that when placing the fibers in the volume, the other fibers are not affected. This ensures that voxels are only replaced if, according to the current estimate, the voxel belongs to a fiber.

## Experiments

To validate the PARE algorithm, two simulation experiments were set up. The numerical phantoms for those experiments were generated from a set of randomly directed fibers and with uniformly distributed centers and lengths. The fiber directions were generated as independent samples drawn from a *von Mises-Fisher* (vMF) distribution. Its probability density function on the sphere for a given direction ${\mathbf {u}} = (\theta , \phi )$ is

12 $$\begin{aligned} f({\mathbf {u}}; {\varvec{\mu }}_{\mathrm{vMF}}, \kappa ) = C_{F} \exp (\kappa {\varvec{\mu }}_{\mathrm{vMF}}^{T}{\mathbf {u}}), \end{aligned}$$

where

13 $$\begin{aligned} C_{F} = \kappa / (4\pi \sinh \kappa ), \end{aligned}$$

and the vector ${\varvec{\mu }}_{\text {vMF}} = (\alpha , \beta )$ denotes the mean direction of the distribution and $\kappa$ the *concentration parameter*, where a large value of $\kappa$ corresponds to a lower variance (i.e., a higher concentration around the mean direction) [24]. The positions of the fibers are drawn from a uniform distribution, with the restriction that each fiber is fully positioned within the volume (i.e., no truncation). The fiber length was $70\pm 10$ vx, also drawn from a uniform distribution. The number of SIRT iterations was set to 100 for all experiments.

**Fig. 5:**
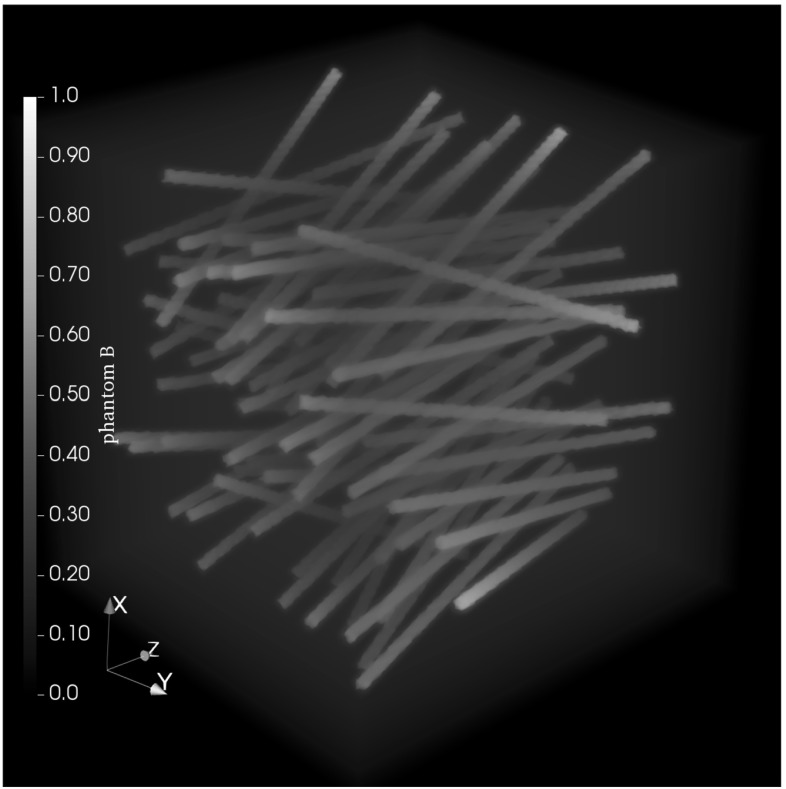
Ground truth of the synthetic phantom B with 72 individual fibers with directions drawn from $f({\mathbf {u}}, {\varvec{\mu }}=(0, 0), \kappa =7)$

We generated two phantoms with $100^3$ voxels, that only differ in the parameters of the direction distribution. For the first phantom, phantom A, the directions were drawn from $f({\mathbf {u}}, {\varvec{\mu }}=(\frac{\pi }{2},0), \kappa =40)$. The second phantom, phantom B, was generated from $f({\mathbf {u}}, {\varvec{\mu }}=(0, 0), \kappa =7)$ (Fig. 5).

With the fibers drawn at random, a version of the Random Sequential Adsorption (RSA) algorithm [25] was performed to generate non-overlapping fibers [26]. To simplify the collision detection in the RSA algorithm, the fibers were treated as sphero-cylinders, reducing the collision problem to finding the closest points of two line segments and a simple distance calculation. To make the fibers behave realistically, the placement of a fiber is also kept if the fibers touch exactly, so if the distance of two fibers is exactly $d_{\overline{1,2}} = r_{1} + r_{2}$. As the aspect ratios of the fibers are high, the error introduced by this approach is negligible.

Due to the higher variance around the mean direction for phantom B, the RSA only placed 72 fibers, while phantom A contains 109 fibers. In both cases the algorithm was initialized to place 150 fibers.

The expected values for both matrix and fiber attenuation were estimated from scans of a real dataset. The background had an intensity of $\mu _{\text {polymer}} = [0.23 \pm 0.07]$ and the fibers had a normalized intensity of $\mu _{\text {fiber}} = [0.76 \pm 0.05]$. Both intensity values are given as percentages of the maximum possible value of the used integer data type. To generate the phantoms we therefore used the value 0.23 for the background and 0.76 for the fibers.

**Fig. 6:**
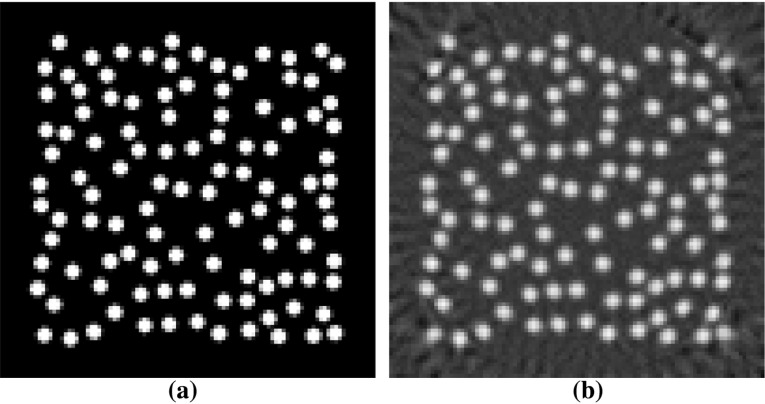
Central slice through the yz-plane of phantom A (**a**) and its reconstruction from simulated projections using 100 iterations of SIRT (**b**). **a** Central slice of phantom A with 109 individual fibers of varying position, direction and length, **b** Central slice of the reconstruction of the phantom shown in (**a**) from 100 simulated projections

From those phantoms we created forward projection images using a simulated cone-beam geometry, as that is the most commonly used geometry in industrial and desktop X-ray scanners. The phantom was placed in the origin of the system. The source-detector distance (SDD) was 250 mm and the source object distance (SOD) was 14 mm. The simulated detector had square pixels with a size of 50 $\upmu$m. This yielded an effective detector pixel size of 2.8 $\upmu$m isotropic in the reconstructions, with a magnification of around 17.86 in the center plane of the phantom. In Fig. 6 the central slice along the yz-plane of a randomly generated phantom and the same slice of a reconstruction of said phantom from simulated projections are shown.

Using the generated data, we evaluated the performance of the PARE method as a function of both the number of projection angles available and the signal-to-noise (SNR). In all cases we chose 100 SIRT iterations as the base line for the reconstructions of the two generated phantoms. We performed one experiment for each noise level $\sigma$ and number of projections, respectively for both phantoms. For the experiments with added noise, we used additive, Gaussian distributed white noise, which was added to the projection data before the reconstruction.

## Results

In this section, the results of the experiments described in the previous section are presented. In particular, the errors in the parameter estimates obtained by PARE are reported. The errors in the length and centroid position coordinates of the fibers were obtained by computing the difference between the estimates of these parameters and their corresponding ground-truth values. The errors in the direction vectors were computed as the angle derived from the dot product of the Cartesian representation of the estimated and ground truth vector, respectively. It should be noted that prior to the computation of these errors, we first have to identify which estimated fiber parameter vector corresponds with which ground truth fiber parameter vector.

To this end, we match each ground truth fiber parameter vector with the vector in the set of estimated fiber parameter vectors that is closest in terms of Euclidean distance. Mathematically, the one-to-one mapping performed can be described as follows. Let the sets of fibers be $F_\mathrm{gt}$ and $F_\mathrm{est}$ the ground truth and estimated fibers, respectively, then the mapping from one set to the other is defined as

14 $$\begin{aligned} f: \forall a_{n} \in F_\mathrm{gt} \mapsto \arg \min \limits _{b \in F_\mathrm{est} \setminus \lbrace f(a_1, \ldots , a_{n-1}) \rbrace } \vert \vert a_{n} - b \vert \vert ^2. \end{aligned}$$

Note that this implies that the mapping depends on the order of processing if two or more fibers from one set have the same distance to one single fiber in the other set. We expect this case to be unlikely and even if it occurs, the error value will presumably be the same for all of them, so the order is not important. If there were less or more fibers detected than are in the ground truth, we only map the ones that fit best and discard the others as not detected. In the former case we only evaluate the error on the fibers that have an estimate associated with them, and in the latter case we find associated fibers for each ground truth fiber and don’t evaluate the rest.

### Performance with Varying Number of Projection Angles

**Fig. 7:**
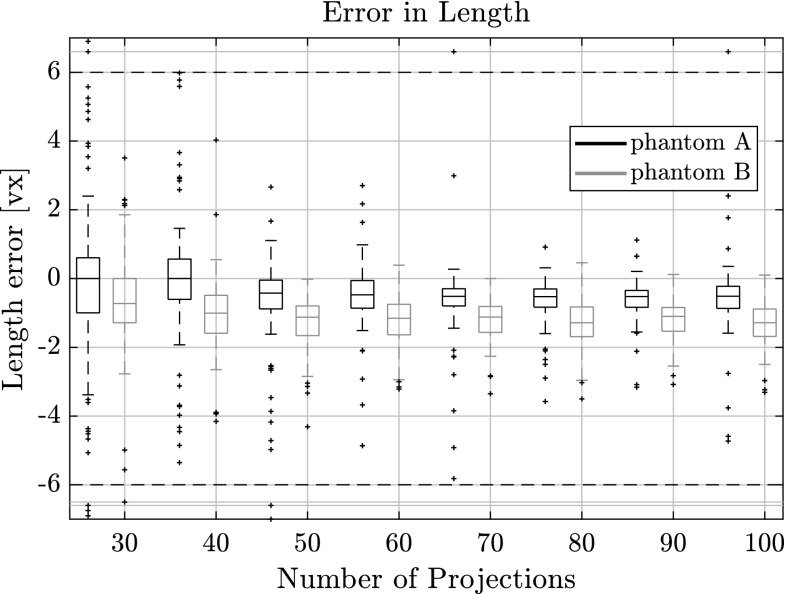
Length error for varying number of projections on phantoms A and B with respect to the estimated fiber length. Outliers were capped at $\pm \,6$ vx, but are still shown outside the horizontal dotted lines

**Fig. 8:**
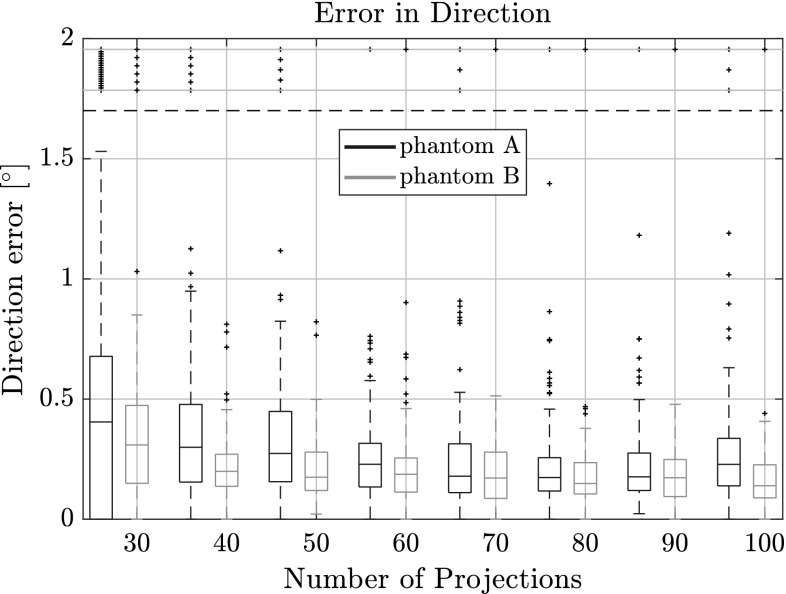
Direction error for varying number of projections on phantoms A and B with respect to the estimated direction vector.Outliers were capped at $2^\circ$, but are still shown outside the horizontal dotted lines

**Fig. 9:**
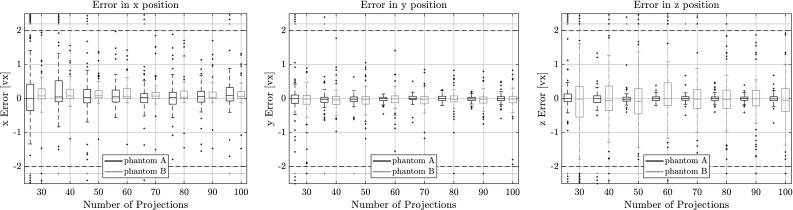
Centroid position error for varying number of projections on both phantoms A and B on the estimated centroid position of the fiber. Outliers were capped at $\pm \,2$ voxels, but are still shown outside the horizontal dotted lines

In Figs. 7, 8 and 9 the quality of the estimation with PARE in function of the number of projections used is shown. In all figures there are two box plots for each projection, where the black one corresponds to results for phantom A and the orange one corresponds to phantom B. It can clearly be seen that the algorithm can retrieve the individual fiber centroids with around $\pm \,0.5$ vx accuracy in the upper and lower quartiles even with as low as 30 projections for both phantoms.

As can be observed from Fig. 9, errors are higher in the coordinate direction that corresponds to the mean axis of the direction distribution. While the direction estimation is not affected by this, the length estimation and centroid estimation are correlated. The length estimation can retrieve the fiber length up to $\pm \,1$ vx for 30 projections. The direction vector can be approximated to about $0.6^\circ$ for the upper quartile. With an increasing number of projections this error naturally decreases, as there is more information available for computing the projection error making the procedure more sensitive to small parameter changes. With 100 projections, the error for the centroid position is as low as $\pm \,0.3$ vx which is around the accuracy of the sub-sampling we do for the voxelization of the fibers in the phantoms. The direction can be estimated to around $0.4^\circ$ for phantom A and $0.25^\circ$ for phantom B. Lengths are estimated between 0.2 and 0.7 vx for phantom A and between 0.9 and 1.8 vx for phantom B.

### Performance in Presence of Noise

**Fig. 10:**
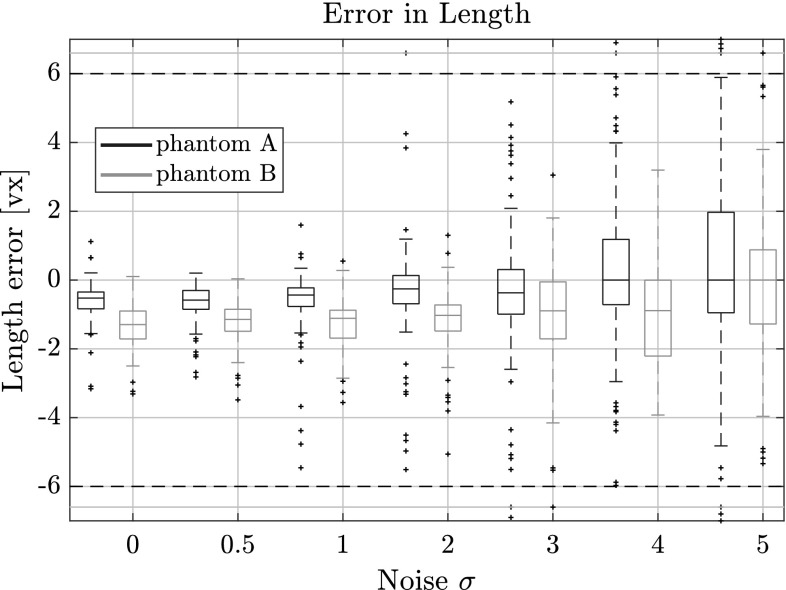
Length error for several noise levels on phantoms A and B with respect to the estimated fiber length. Outliers were capped at $\pm \,6$ vx, but are still shown outside the horizontal dotted lines

**Fig. 11:**
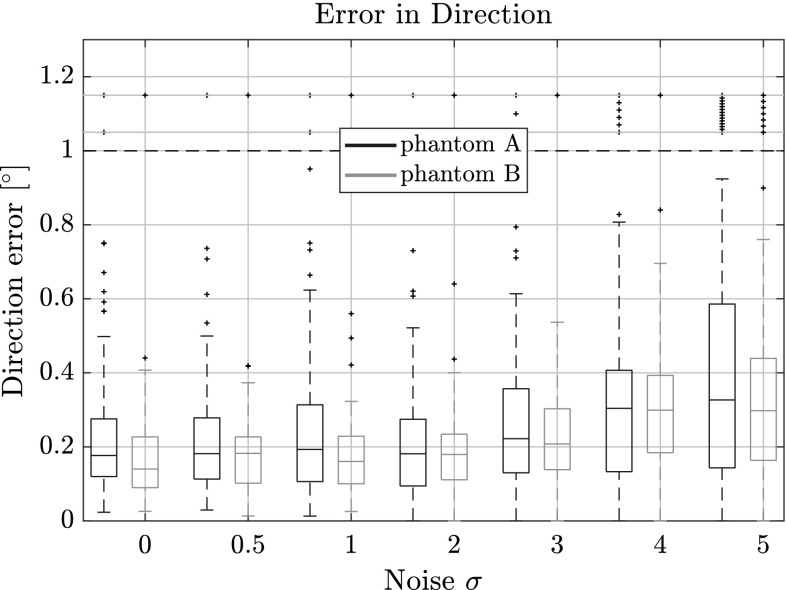
Direction error for for several noise levels on phantoms A and B with respect to the estimated direction vector. Outliers were capped at $1^\circ$, but are still shown outside the horizontal dotted lines

**Fig. 12:**
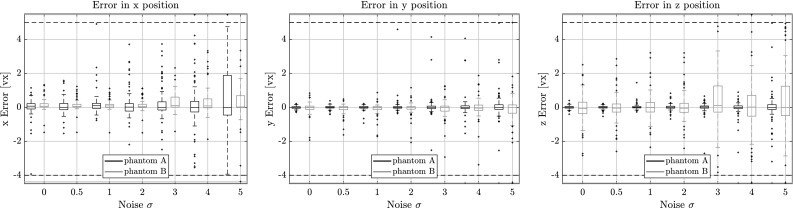
Centroid position error for for several noise levels on both phantoms A and B on the estimated centroid position of the fiber. Outliers were capped at $\pm \,4$ voxels, but are still shown outside the horizontal dotted lines

In Figs. 10, 11 and 12 the length, direction and position errors are shown in function of the standard deviation $\sigma$ of additive noise we added to the projection data. As expected, the errors increase with increasing $\sigma$. The length estimates are barely changing for the lower noise levels $\sigma = 0.5$ and $\sigma = 1.0$ and are in the same range as the errors for 100 projections in the previous tests. This is also expected, as we used 100 projections consistently for this experiment. The signal-to-noise ratio (SNR) for the different noise levels and phantoms are laid out in Table 1. We compute it by

15 $$\begin{aligned} \mathrm{SNR = 10 \log } \left( \frac{\mu _\mathrm{signal}}{\sigma _\mathrm{noise}} \right) , \end{aligned}$$

where $\mu _\mathrm{signal}$ is the mean of the measured intensity of the projections and $\sigma _\mathrm{noise}$ the corresponding noise level.

The length and the centroid estimates seem to be more affected by the noisy projections than the direction estimates. In the case of the highest noise level, the length estimate is 2 vx too large in the upper quartile for phantom A and around 1 vx for phantom B.

This is most likely due to the way voxels change in the simulated projections of our model. When varying the direction vector, more voxels change their value, compared to when the length or centroid position is changed. This in turn means that the optimization is more sensitive to small changes in direction, especially when the fibers are very long.

**Table 1:** SNR for the noise levels used in our experiments for both phantoms

$\sigma$	$\mathrm SNR_\mathrm{A} (dB)$	$\mathrm SNR_\mathrm{B} (dB)$
0.5	17.06	16.88
1.0	14.05	13.87
2.0	11.04	10.86
3.0	9.27	9.10
4.0	8.03	7.85
5.0	7.06	6.88

## Discussion

Our algorithm’s main advantage is the use of a parametric fiber model of which the parameters are estimated directly in the projection domain, thereby largely avoiding reconstruction artifacts that may otherwise influence the fiber position and direction estimation. As a result, the parameter estimation is robust even for a very small number of projections. Most algorithms trying to estimate fiber parameters use several thousands of projection images to compute quantities on their fiber specimen [4, 7, 10].

However, Parametric Reconstruction (PARE) is limited by a couple of factors. The rigid cylinder model is adequate for fibers that are not bent, which is a reasonable assumption in GFRPs that have moderate aspect ratios. In case of high aspect ratio fibers, the model would need to be extended to allow bending. Altendorf and Jeulin proposed an approach to model fibers as short fiber segments on a chain and generated random fiber networks from it using a random walk approach [27]. A similar model, of cylinders chained together for example, could be used to represent the fibers in our approach, but would require heavy modification of the Hough transform or a different approach for the clustering of detected fiber center lines altogether. As the Hough transform can be defined for an arbitrary parametrized curve [28], the model could be transformed to approximate weaving in carbon fibers for example. This of course would increase the number of parameters in the Hough accumulator exponentially and therefore might not be practical for very complicated fiber systems.

In the future we plan to iteratively refine the model during the SIRT reconstruction giving the estimates a feedback mechanism to improve over time. As a first step we intend to incorporate new discrimination schemes like the one proposed in [29] as at the moment crossing fibers pose a problem with the detection using the Hough transform, giving falsely detected fibers. This will be a first step towards making the method ready for use with real world data, which will be important to evaluate its performance in practical applications. In the process of this future work, the model will most likely be adapted as well, as the method in itself is invariant to which model is used.

## Conclusion

A new method, Parametric Reconstruction (PARE), was presented. The method detects fibers in a reconstructed volume, represents those fibers with a parametric model and optimizes their centroid position, direction and length using the projection distance as a reference. The method was shown to accurately estimate the fiber parameters direction, centroid position and length in synthetic data. It was also shown that the estimation can recover the parameters of the fibers even with less than 100 projections available, as well as on very noisy data.
